# 

**Published:** 1900-06-09

**Authors:** 


					THE HOSPITAL. <? The standard of highest purity." ?Lancet. June 9tii, 1900.
CADBURY'S if U ? CADBURY'S
? M JEL. COCOA
cocoa m NO DRUGS OR ALKALIES USED.
ABSOLUTELY PVIX.
No. 715. Vol. XXVIII. UKS1S Saturday,
June 9, 1900.
^Subscriptf on Ijerms,] pRICE TWOPENCE.

				

